# Medium-Sized Highly Coupled Planar Arrays with Maximum Aperture Efficiency

**DOI:** 10.3390/s21175925

**Published:** 2021-09-03

**Authors:** Peyman Pourmohammadi, Vladimir Volski, Guy A. E. Vandenbosch

**Affiliations:** ESAT-WAVECORE Research Division, Katholieke Universiteit Leuven (KU Leuven), Kasteelpark Arenberg 10, 3001 Leuven, Belgium; peyman.pourmohammadi@esat.kuleuven.be (P.P.); vladimir.volski@esat.kuleuven.be (V.V.)

**Keywords:** antenna array, aperture efficiency, Internet of Things (IoT), multi input-multi output (MIMO)

## Abstract

This paper presents a technique to design strongly coupled planar arrays with very high aperture efficiency. The key innovation is that, based on an irregular 2 × 1 array, very compact medium-sized arrays of size 2 × 2, 2 × 4, and 2 × 6 are constructed with very strong and constructive mutual coupling between the elements. In this way, a maximum aperture efficiency is reached for a given footprint of the array. The occupied space of the antenna in comparison with conventional linear patch arrays is studied. A prototype 2 × 4 array operating around 5.8 GHz is designed, fabricated, built, and measured. The results show a large bandwidth of 20% and a very high aperture efficiency of 100%, which is the largest found in the literature for similarly sized arrays. These results are important in view of the future Internet of Things, where small and medium-sized arrays are planned to be mounted on numerous devices where a very limited physical area is available.

## 1. Introduction

During the last decade, wireless devices such as mobile phones, navigation systems, etc., have become an intimate part of our daily life. Hence, antennas as one of the key components in wireless communication systems have received a lot of attention. Planar (patch) antennas are one of the attractive candidates for wireless communications, thanks to the lightweight, low profile, low cost, easy fabrication, and ability to integrate with microwave circuits [1].

In many cases, it is required to design antennas with high gain. One of the easiest ways to increase gain is to use an array structure [1]. However, due to the physically limited space in small devices, the typically available footprint of the antenna is limited and the array has to be miniaturized. High gain for a small footprint means a high aperture efficiency [2]. Aperture efficiency is a well-known quantity in the field of antennas. However, since it is the core concept of this paper, for completeness we repeat its definition here. The aperture efficiency of an antenna is the ratio of the effective receiving area of an antenna to the physical area of the antenna, see Equation (1). In other words, this quantity describes how effective an antenna is receiving power [2]. It is directly related to the gain of the antenna, see Equation (2).

(1) $$\eta \;=\frac{A_{\mathrm{eff}}}{A}$$

(2) $$A_{\mathrm{eff}}=\frac{\lambda^{2\;}\times \;G}{4\pi }$$

where A is the physical area of the antenna, λ is the wavelength in free space, and G is the gain of the antenna. Note, since the directivity is not affected by losses in the antenna, the gain is considered instead of directivity in Equation (2). The antenna gain is the product of directivity and efficiency.

The aperture efficiency of medium-sized arrays is a topic that has not been studied that much in the literature. In [3] a microstrip antenna array has been reported with 90% aperture efficiency. However, this antenna has a very narrow bandwidth, which does not meet the requirements of today. A quite complex broadband printed compound air-fed array antenna was presented in [4]. The reported aperture efficiency and bandwidth are 71% and 9%, respectively. A first attempt to design medium-sized high aperture efficiency arrays is reported in [5,6]. The bandwidths reached are around 10% and the aperture efficiencies around 50–60%. An improved series-fed crossed 2 × 2 array was designed in [2]. The aperture efficiency and bandwidth are 84% and 10%, respectively. Recently, a patch antenna with both sides shorted in [7] achieved a wide bandwidth of 13% and an aperture efficiency of about 78%.

It is worth mentioning that a major parameter determining the size of an array is the spacing between adjacent elements. Reducing the spacing to decrease the footprint increases the mutual coupling between the elements [8]. Many methods are available in the literature to reduce mutual coupling, for example using Electromagnetic Band Gap (EBG) structures [9,10,11], Defective Ground Structures (DGS) [12,13,14], Waveguide Metamaterials (WG-MTMs) [15,16], and parasitic elements between the antennas [17,18]. However, none of these techniques will be used here.

Instead, in this paper, the mutual coupling will be constructively used as a tool to reduce the footprint of the antenna, and thereby increase the aperture efficiency, while keeping a considerable bandwidth, as required by modern-day applications. The paper is organized as follows. First, in Section 2 a compact 2 × 1 basic building block is proposed. This block is prototyped and characterized. In Section 3, Section 4 and Section 5 arrays of size 2 × 2, 2 × 4, and 2 × 6 are described, respectively. An overview table is given comparing the results reached with results available in the open literature. Finally, conclusions are drawn in Section 6.

## 2. Basic 2 × 1 Array Building Block

The design procedure begins by selecting a basic 2 × 1 array that is going to be used as a basic block to build the larger arrays.

**Figure 1:**
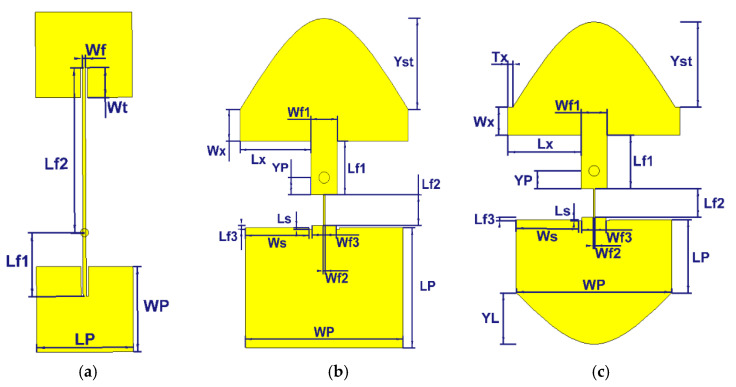
Topologies: (**a**) preliminary patch antenna array; (**b**) proposed patch antenna array with rectangular bottom patch; (**c**) proposed patch antenna array with two elliptical patches.

The main idea is to start from a traditional 2 × 1 rectangular patch array fed along a microstrip line connecting the two patches in such a way that the patch currents are in phase, see Figure 1a. Next, the strategy is to modify the shape of the patches and microstrip line in such a way that the in-phase current is maintained as much as possible, but with a much smaller footprint. This can be achieved by constructively using the stronger mutual coupling between the two modified elements. This system resembles the general case of two coupled resonators, where the resonant frequencies are known to be (strongly) affected by the coupling. The main challenge is to design the two resonant frequencies in the presence of strong mutual coupling so that their combination matches the band that is targeted. This is achieved in this paper by full-wave simulations.

Essentially, three steps have been taken to reach this goal. First, the length of the transmission lines is decreased to reduce the footprint. Second, the shape of the upper element is chosen. It should be noted that many different shapes have been studied. The best result obtained (large bandwidth, small footprint) was with half an elliptical shape for the upper patch. Third, in order to have 50 Ω impedance matching tuned microstrip lines are used with optimized widths. The resulting structure can be seen in Figure 1b.

It is worth emphasizing that the two patches should be as close as possible in order to achieve a high aperture efficiency. To bring about this goal, the length of the microstrip line connecting the two patches needs to be decreased. Conventionally, the transmission line length needs to realize the required phase difference between the patches, which is at first sight, impeding changing this length. Only the strong mutual coupling when shortening the line makes this possible. This coupling has a very complex nature and can be monitored by looking at S-parameters and current distribution. Additional requirements for this line are that the antenna should be matched, and both patches should be excited.

In order to validate the concept two prototypes operating at 5.8 GHz were designed (using CST Microwave Studio, version 2019, Dassault Systèmes, Vélizy-Villacoublay, France), fabricated, and measured, one without an air layer, resulting in a smaller bandwidth, and one with an air layer, resulting in a larger bandwidth. Rogers 4003C (Rogers Corporation, Ghent, Belgium) is used as a substrate, with a thickness of 0.813 mm, permittivity 3.38, and loss tangent 0.002. All dimensions (both for the preliminary structure and the two miniaturized building blocks, without and with an air layer) are given in Table 1, the first three columns.

### 2.1. Building Block without Air Layer

**Figure 2:**
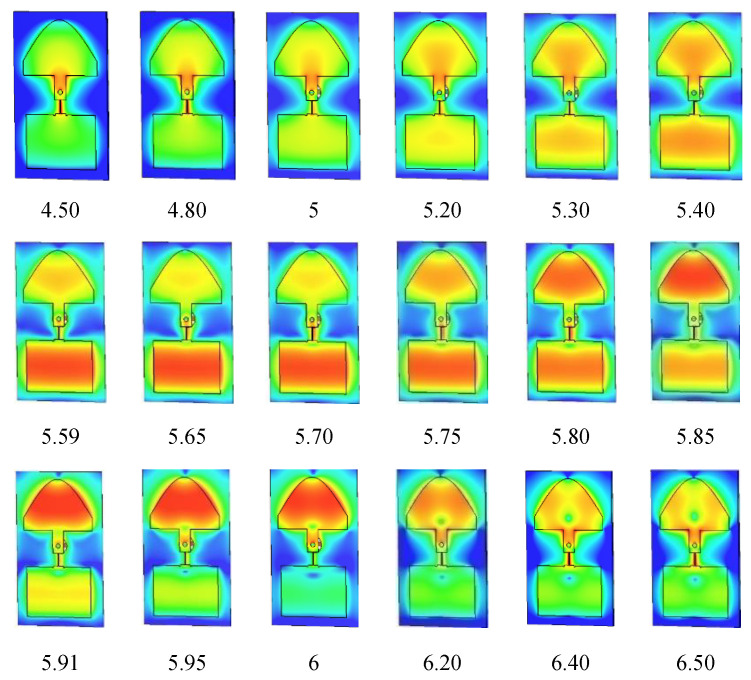
Current distributions on the proposed microstrip array as a function of frequency (in GHz).

**Figure 3:**
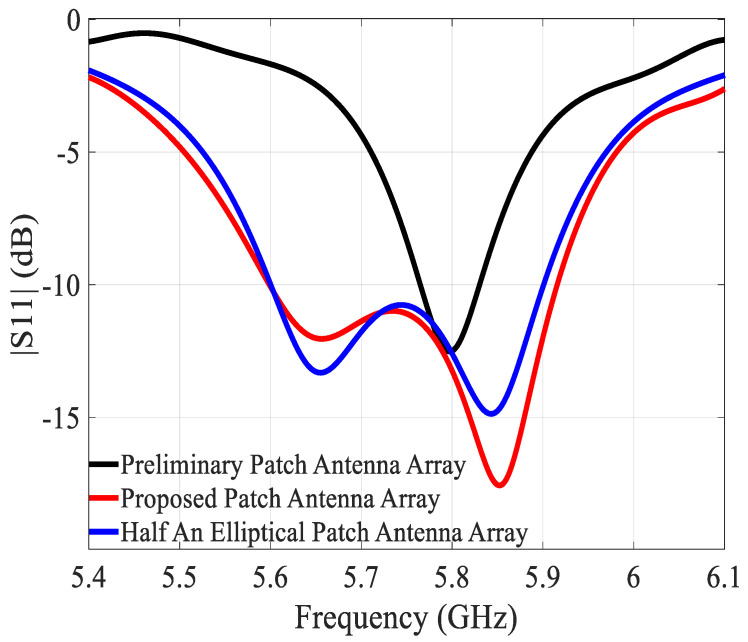
Simulated |S11|of the preliminary array, proposed array with rectangular bottom patch, and proposed array with two half elliptical patches.

Figure 2 shows the surface current distribution as a function of frequency. It is perceived clearly that a first resonance at a lower frequency (5.65 GHz) occurs in the bottom rectangular patch, followed by a second resonance at a higher frequency (5.85 GHz) in the half elliptical patch. At 5.8 GHz, the intensity of the current is almost the same in the two patches. This combination of resonance frequencies, in combination with and affected by the strong mutual coupling, effectively expands the overall bandwidth. Concerning the effect of the shape of the bottom patch, it can be noted that an almost identical evolution of the current with frequency is found in the case of the structure with two half-elliptical patches (see Figure 1c). There is only a very small difference between the two cases, as is illustrated also in Figure 3 where the simulated reflection coefficients are given. The elliptical shape for the bottom patch yields a bandwidth which is only 20 MHz smaller than the structure with a rectangular bottom patch. This means that the shape of the lower patch is not that crucial. Almost the same results can be obtained with other shapes, following the same procedure.

For the preliminary array, the miniaturized array with rectangular bottom patch, and the miniaturized array with two half elliptical patches, the −10 dB bandwidths are 70 MHz (5.76–5.83 GHz), 320 MHz (5.59–5.91 GHz), and 300 MHz (5.59–5.89 GHz), respectively. The gains at 5.8 GHz are 7.2, 7.5, and 7.1 dBi. The aperture efficiencies at 5.8 GHz are 89%, 112%, and 100%, and the radiation efficiencies at 5.8 GHz are 77%, 83%, and 82%, respectively. It is worth pointing out that an aperture efficiency greater than 100 % just means that electrically the antenna is larger than its physical size [1].

**Figure 4:**
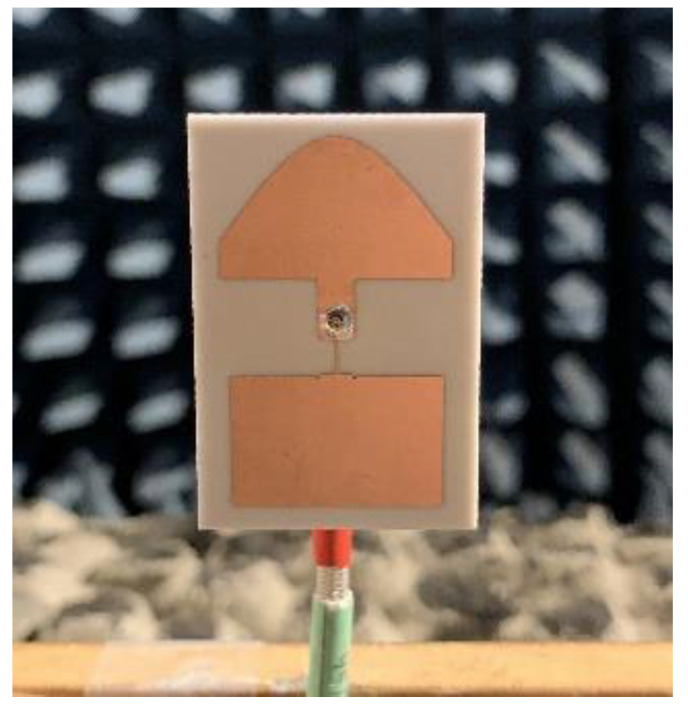
Prototype of the 2 × 1 building block.

**Figure 5:**
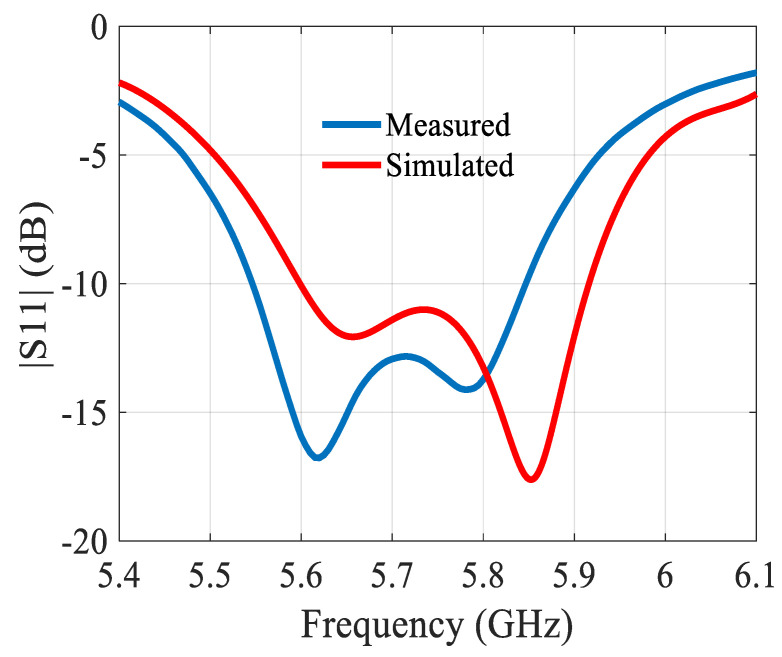
Reflection coefficient of the 2 × 1 building block.

The preliminary array was simulated on a 1.15λ × 0.41λ ground plane, and the two proposed miniaturized arrays were simulated on a 0.81λ × 0.5λ ground plane. The built prototype is shown in Figure 4. Of note is the SMA connector feeding the microstrip line of the antenna. Figure 5 depicts the reflection coefficient, showing a difference of ca. 60 MHz between simulated and measured operating frequency.

**Figure 6:**
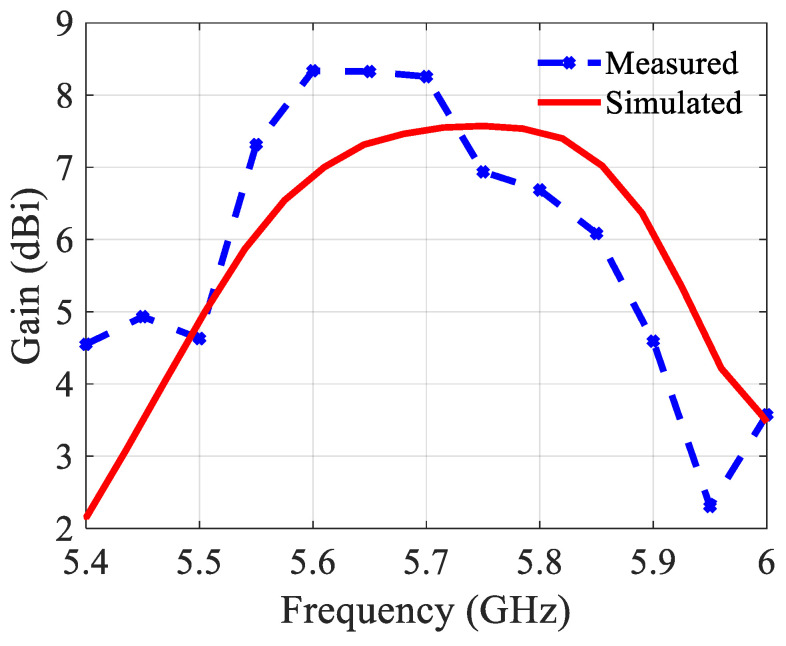
Gain of the 2 × 1 building block.

**Figure 7:**
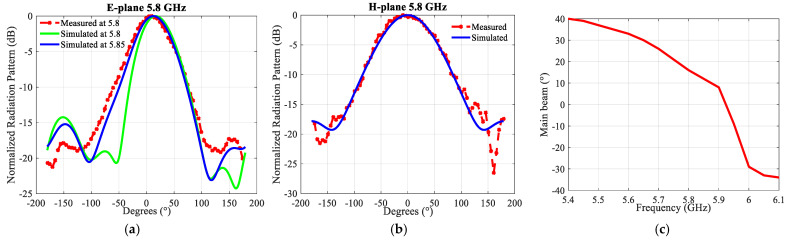
Simulated and measured normalized radiation patterns of the 2 × 1 building block at 5.8 GHz: (**a**) in the E-plane; (**b**) in the H-plane; (**c**) simulated direction of the main beam in the E-plane.

Figure 6 depicts the gain and Figure 7 the radiation patterns. In the H-plane, the simulated and measured radiation patterns agree well. In the E-plane, the direction of the main beam varies over the bandwidth, see Figure 7c. The change in the main beam direction with frequency is a typical feature of this antenna. It is linked with the fact of the intrinsic use of the mutual coupling.

Taking into account the shift in|S11| in Figure 7a the simulated radiation pattern in the E-plane at 5.85 GHz is also depicted. It agrees extremely well with the radiation pattern measured at 5.8 GHz. The radiation patterns are compared at the frequency of resonance, which is slightly different in simulations and measurements. The antenna has −3 dB beamwidths of ~57° and ~86° in the E- and H-plane at 5.8 GHz, respectively. The differences between simulated and measured values are small enough so that they can be entirely attributed to the tolerances on the permittivity of the substrate, and the dimensional precision of the fabricated antenna.

### 2.2. Building Block with Air Layer

**Figure 8:**
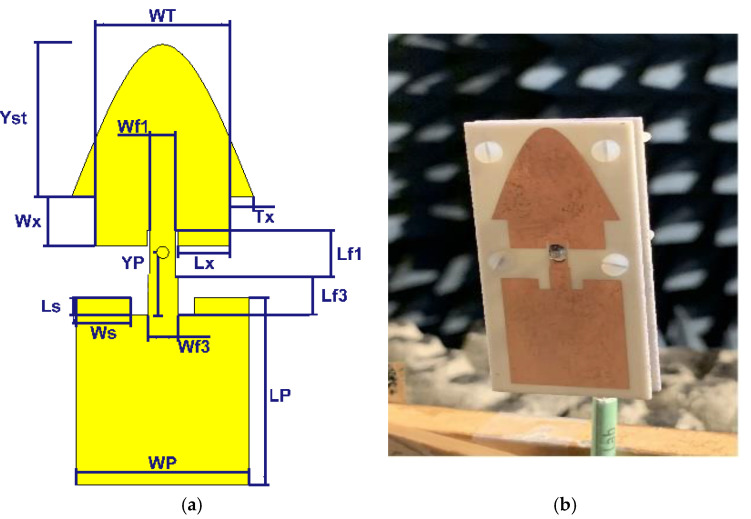
Configurations: (**a**) the 2 × 1 building block with an air layer (**b**) prototype of the 2 × 1 building block with an air layer.

**Figure 9:**
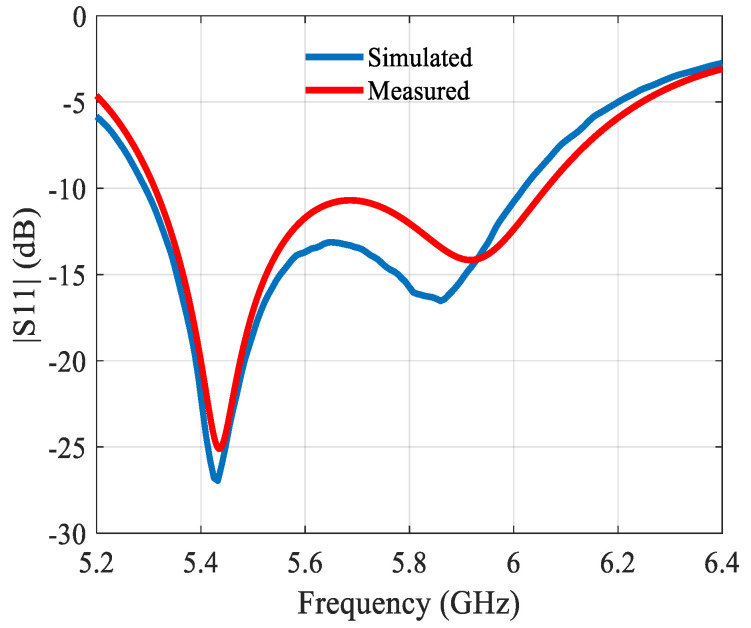
Reflection coefficient of the 2 × 1 building block with an air layer.

**Figure 10:**
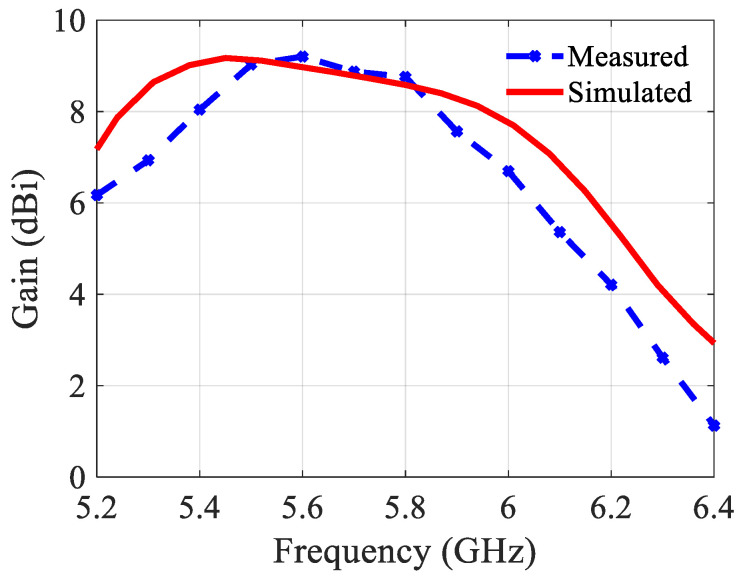
Gain of the 2 × 1 building block with an air layer.

**Figure 11:**
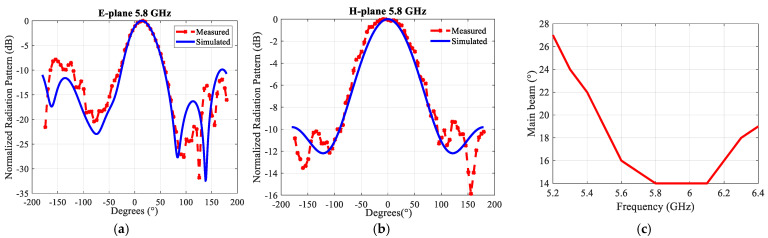
Simulated and measured normalized radiation patterns of the 2 × 1 building block with an air layer at 5.8 GHz: (**a**) In the E-plane; (**b**) In the H-plane; (**c**) Simulated direction of the main beam in the E-plane.

The slightly modified topology and the built prototype are shown in Figure 8. The structure includes two substrate layers (same type) with an air gap in between. The radiating part is on the top substrate. An SMA connector is mounted on the back of the second substrate. Nylon (polyamide) spacers of 3 mm long realize the 3 mm air layer. The reflection coefficient is shown in Figure 9. The measured results agree well with the simulated ones. The bandwidth is 730 MHz (5.34–6.07 GHz) or 12%, which is more than double compared to the structure without an air layer. The gain, the aperture efficiency, and the radiation efficiency at 5.8 GHz are 8.6 dBi, 115%, and 98%, respectively. Figure 10 depicts the gain and Figure 11 the radiation patterns.

As shown in Figure 11, the measured and simulated radiation patterns are in a good agreement. There are different values in the backside direction (+180/−180) due to the measurement equipment. The antenna has to be rotated on the setup depending on the measurement plane (E or H). As a consequence, the orientation of the feeding cable is not the same for both measurement topologies, resulting in different levels in the backside direction. Figure 11c shows the main beam’s direction in the E-plane changing across the bandwidth. The variation is much less than in case of the structure without an air layer, and from 5.8 GHz up to 6.1 GHz this beam direction is quasi constant at ca. 14°.

## 3. 2 × 2 Array

In the previous section, by choosing a proper topology for the 2 × 1 array in the E-plane, and thus using mutual coupling in a constructive way, we were able to maximize the aperture efficiency of the basic building block. In this section, the effect of putting two building blocks in each other’s H plane will be studied in order to see whether, also in this plane, the miniaturization strategy still works.

**Figure 12:**
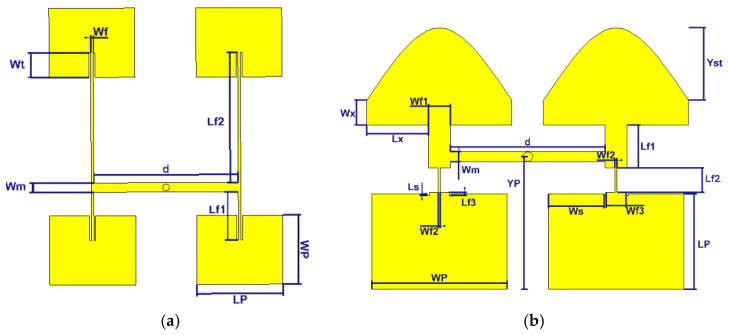
Configuration of the (**a**) conventional 2 × 2 antenna array (**b**) proposed 2 × 2 antenna array.

Following the same line of reasoning as in the previous section, the geometry of the conventional 2 × 2 array and the proposed 2 × 2 array are depicted in Figure 12.

An actual design was made, based on the conventional and proposed building block without an air layer of the previous section, while optimizing the distance d and slightly further optimizing the dimensions to reach the best compromise for large bandwidth and large gain. All dimensions of the resulting 2 × 2 arrays are detailed in Table 1. Note that the distance d is slightly smaller than for the preliminary 2 × 1 array.

**Figure 13:**
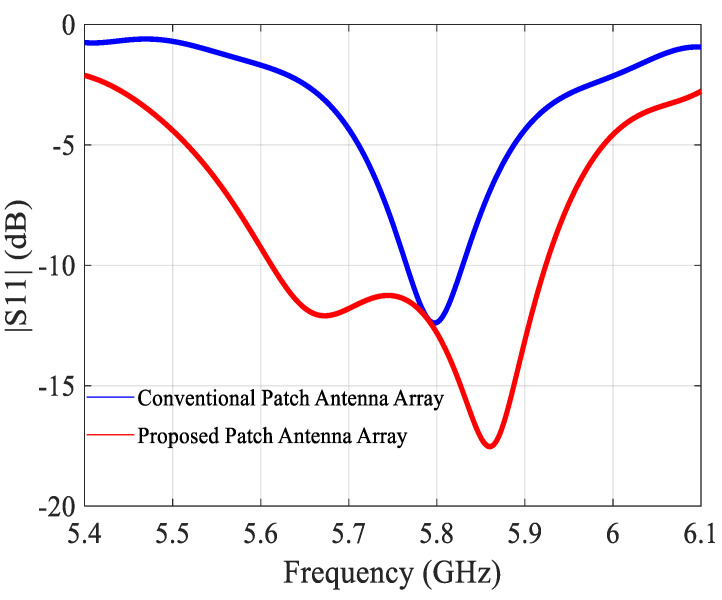
Simulated |S11| of the conventional and proposed 2 × 2 antenna array.

**Figure 14:**
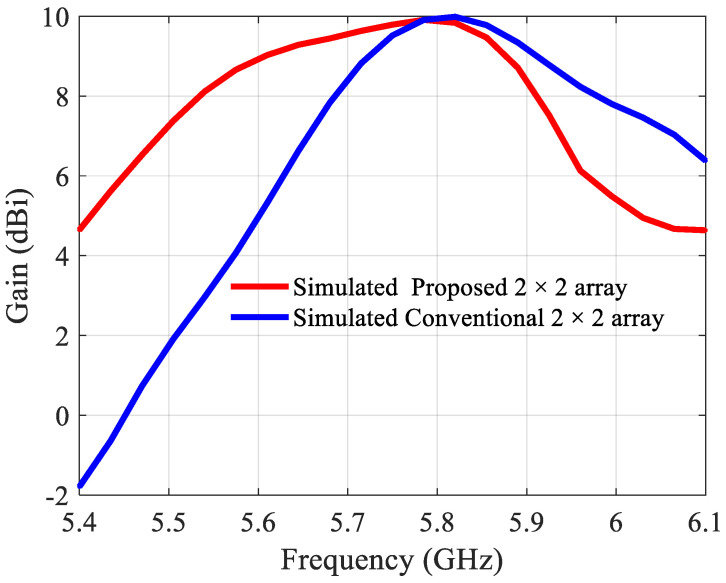
Simulated gain of the conventional and proposed 2 × 2 antenna array.

The simulated |S11| of the conventional and the miniaturized 2 × 2 array are shown in Figure 13. The −10 dB bandwidths are 70 MHz (5.75–5.82 GHz) and 340 MHz (5.56–5.90 GHz), respectively, which is similar as for the 2 × 1 arrays. The gains are both 9.9 dBi at 5.8 GHz (Figure 14), the aperture efficiencies at 5.8 GHz are 70% and 97%, and the radiation efficiencies at 5.8 GHz 80% and 87%, respectively.

**Figure 15:**
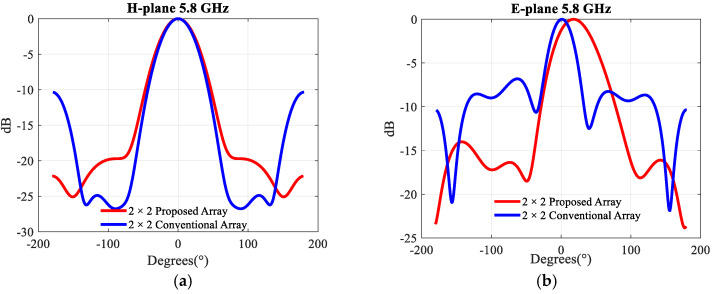
Simulated radiation patterns in (**a**) the H-plane, (**b**) the E-plane of the 2 × 2 conventional and proposed arrays at 5.8 GHz.

Note that the conventional array was simulated on a 1.14λ × 0.98λ ground plane, and the proposed 2 × 2 array on a 0.99λ × 0.81λ ground plane. The radiation patterns at 5.8 GHz are given in Figure 15. The conventional and the proposed 2 × 2 array behave similarly in the H-plane. Both of them have a −3 dB beamwidth of ~50°. In the E-plane, these beamwidths are 35° and 57°, respectively. The direction of the main beam at 5.8 GHz in the E-plane of the proposed 2 × 2 array is ~20°. These results validate the miniaturization scheme in both the E and H plane. This opens a very interesting way to increase the size of the array further by adding extra elements. This approach is examined in the next section.

## 4. 2 × 4 Array

**Figure 16:**
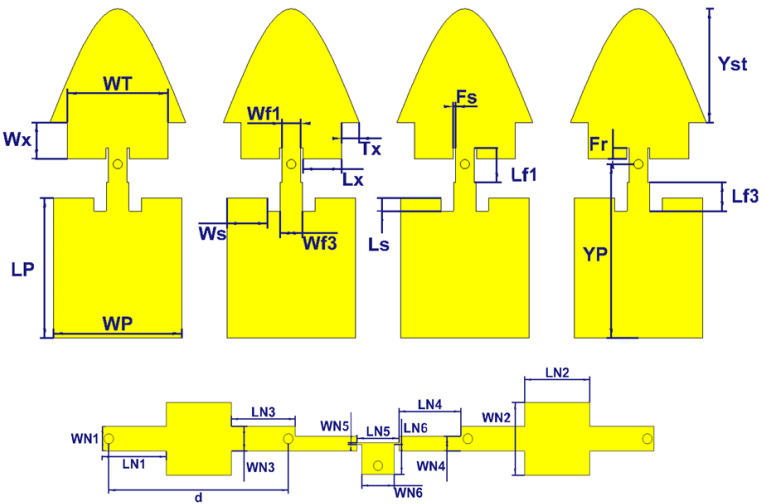
Configurations of the proposed 2 × 4 antenna array with the feeding network.

In the previous section, the performance of an array of two building blocks without an air layer in each other’s H-plane was studied. It was shown that the miniaturization strategy worked well and that the structure has maximum aperture efficiency. In this section, we use four building blocks with an air layer in the array. The building block with an air layer is further optimized slightly in order to match to the feeding network of the array, see Figure 16. All dimensions of the optimized structure are stated in Table 1.

The four 2 × 1 building blocks are fed by four conductive pins with a diameter of 1.36 mm transferring the signal equally with the same amplitude and phase from a feeding network in the bottom metal layer to the top radiating layer.

**Figure 17:**
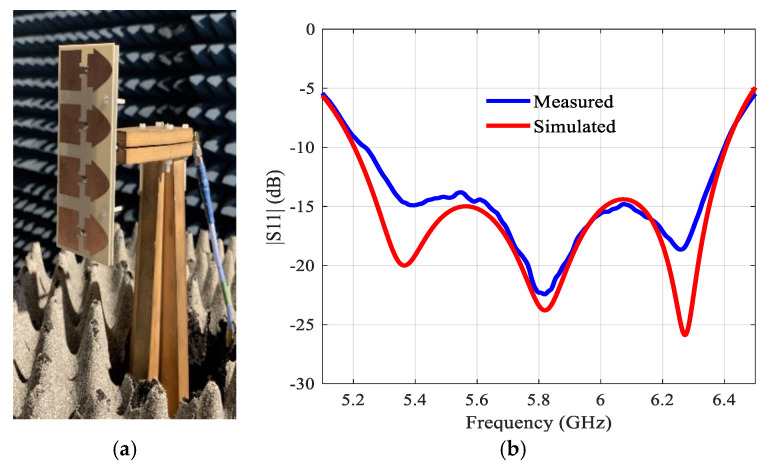
(**a**) Prototype of the 2 × 4 array, (**b**) Simulated and measured reflection coefficients (|S11|) of the integrated structure including the feeding network.

The antenna array was prototyped on a 1.94λ × λ ground plane. The fabricated prototype is Figure 17a. The return loss was measured and is compared with the simulation in Figure 17b. The agreement is quite good. The simulated bandwidth (|S11| ˂ 10 dB) is 5.2 to 6.4 GHz (1.2 GHz).

**Figure 18:**
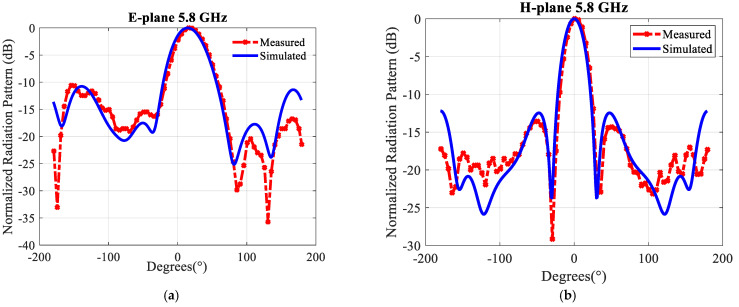
Simulated and measured normalized radiation patterns of the 2 × 4 proposed antenna array with an air layer at 5.8 GHz: (**a**) In the E-plane (**b**) In the H-plane.

**Figure 19:**
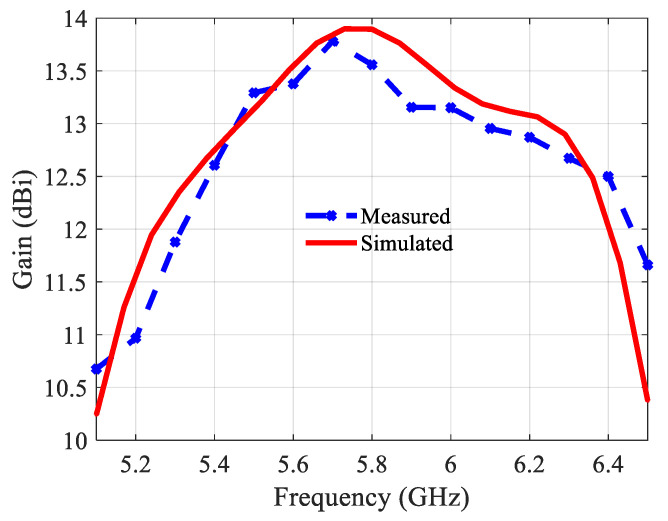
Simulated and measured gains.

Figure 18 depicts the far-field radiation patterns of the antenna array in the E- and H-plane at 5.8 GHz. The measured results agree well with the simulated results. The proposed antenna has a 3-dB beamwidth of ~43° and 26° in the E- and H-plane at 5.8 GHz, respectively. The variation in the gain, both simulated and measured, over the matching bandwidth, as shown in Figure 19, is about 1 dB, which is very satisfactory. The maximum gain in simulation is 13.9 dBi at 5.8 GHz. Aperture efficiency and radiation efficiency are 100% and 96% at 5.8 GHz, respectively. It has to be emphasized that losses were considered in the simulations.

These results verify that by increasing the size of the array with an air layer in the H-plane, the proposed antenna array yields a larger bandwidth with a very high aperture efficiency. In the next section, the forming of a MIMO system is investigated.

## 5. 2 × 6 Array

**Figure 20:**
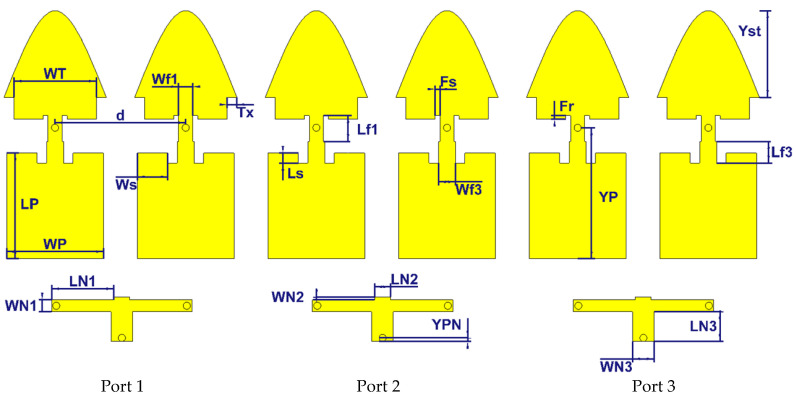
Configurations of the proposed 2 × 6 antenna array with the feeding network.

In this section, the objective is to study a medium-sized MIMO system. This system can be achieved by putting six optimized building blocks with an air layer with six conductive pins feeding them. Three pairs of two neighboring building blocks are combined into three 2 × 1 subarrays, in this way forming a MIMO system with three input-output ports. Each 2 × 1 subarray is thus fed by one 50 Ω SMA connector and a small feeding network, see Figure 20. The overall dimensions are 2.95λ × λ. The detailed dimensions are given in Table 1.

**Figure 21:**
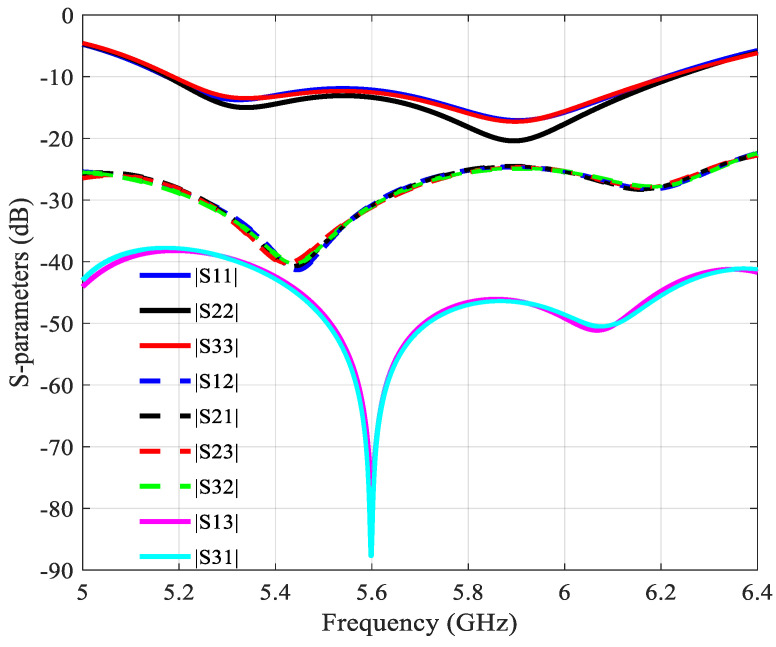
Simulated S-parameters of the 2 × 6 array with 3 ports.

The simulated S-parameters of the array are shown in Figure 21. The simulated −10 dB bandwidth is from 5.15 to 6.18 GHz, or about 17% for a center frequency of 5.8 GHz. A low mutual coupling between the three ports is observed, below −25 dB in the whole operation band.

**Figure 22:**
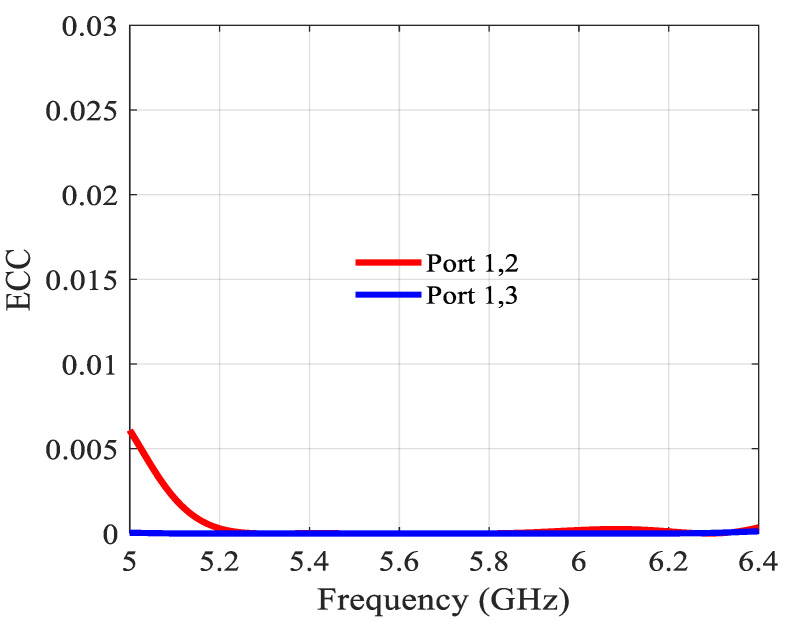
ECC of the 2 × 6 MIMO array.

The envelope correlation coefficient (ECC), an important parameter in MIMO antenna systems that characterizes the diversity of the patterns, is plotted in Figure 22. The ECC is below 0.005 in the whole operation band, which is much lower than the still acceptable value of 0.5 [19].

**Figure 23:**
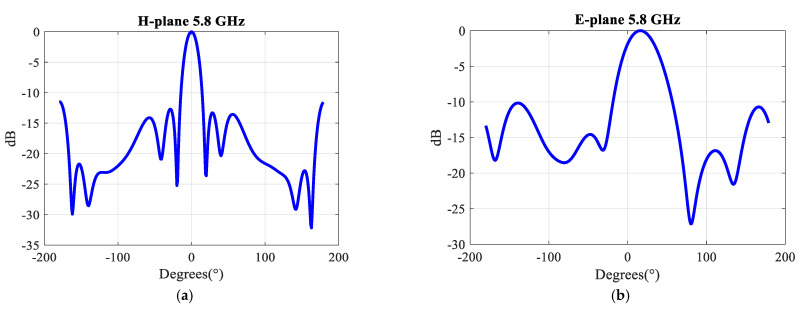
Simulated radiation patterns in (**a**) the H-plane, (**b**) the E-plane of the 2 × 6 array at 5.8 GHz in the case of in-phase excitation of the three MIMO ports.

**Figure 24:**
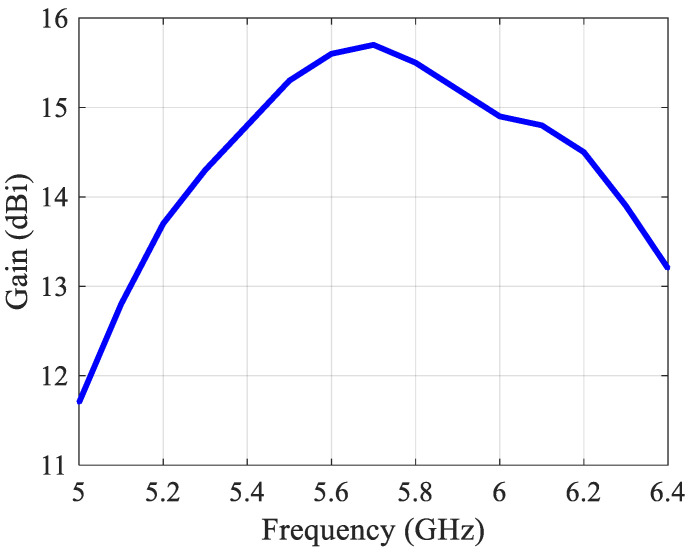
Simulated gain of the 2 × 6 array in the case of in-phase excitation of the three MIMO ports.

In order to illustrate the reachable directivity of this array, the simulated radiation patterns for a uniform excitation of the three ports in the E- and H-plane at 5.8 GHz are shown in Figure 23. The −3 dB beamwidth in the H plane is 17°. The simulated gain is 15.53 dBi, see Figure 24. At 5.8 GHz the radiation efficiency is 96% and the aperture efficiency is also 96%.

## 6. Comparison with Literature

In Table 2 the performance of the proposed antenna arrays is compared with the results described in [2], also a paper focusing on aperture efficiencies. It is clearly seen that the proposed antenna arrays have a higher aperture efficiency and bandwidth for similar radiation efficiencies. However, on top, the design procedure in this paper is focusing on antennas, where small and medium-sized arrays will have to be mounted on all sorts of devices with a very limited available physical area. Taking into account the topological situation in a typical room, therefore radiation patterns are targeted with a smaller (possibly steerable) horizontal beamwidth and a somewhat larger vertical beamwidth. The design procedure yields an excellent MIMO performance, as illustrated in the previous section.

## 7. Conclusions

In this paper, wideband compact medium-sized arrays using mutual coupling in a constructive way have been presented. The antennas have a quite simple structure and reach the highest aperture efficiencies reported in the literature. In combination with their radiation pattern behavior, they are ideal candidates to be used in 5G IoT applications, where they can be mounted on all sorts of devices with a small physical area available.

## Figures and Tables

**Table 1:** Dimensions (Pre = Preliminary, Prop = Proposed with bottom rectangular patch, Hellip = with two half elliptical patches, Conv = Conventional).

Parameter Name	Pre 2 × 1	Prop 2 × 1	Hellip 2 × 1	Prop 2 × 1	Conv	Prop	Prop 2 × 4	Prop 2 × 6
				(Air Layer)	2 × 2	2 × 2	(Air Layer)	(Air Layer)
WP	13.47	19.39	18.95	19.1	13.28	19.39	19.1	19.1
LP	15.24	13.66	8.95	20.84	16.5	13.66	20.84	20.84
Wf1	0.5	3.28	3.16	2.8	0.5	3.17	2.8	2.8
Lf1	10.04	6.12	6.5	5.1	9.23	6.15	5.1	5.1
Wf2	0.5	0.22	0.22	-	0.5	0.29	-	-
Lf2	26.03	3.49	3.49	-	24.97	3.49	-	-
WT	4.7	-	-	15.01	4.7	-	15.01	16.26
Wf3	-	2.96	2.98	3.34	-	2.86	3.34	3.34
Lf3	-	0.44	0.41	4.27	-	0.46	4.27	4.27
Ws	-	7.84	7.66	6.02	-	7.97	6.02	6.02
Ls	-	0.2	0.11	1.97	-	0.2	1.97	1.97
Wx	-	3.59	3.44	5.42	-	3.56	5.42	5.42
Lx	-	8.72	8.93	5.78	-	8.81	5.78	5.78
YP	-	1.96	2.18	7	-	19.01	25.85	25.85
Yst	-	10.37	10.43	17.09	-	10.39	16.9	17.1
Tx	-	-	0.57	2.6	-	-	2.6	1.97
Wm	-	-	-	-	1.8	1.42	-	-
d	-	-	-	-	27.73	25.34	25.8	25.8
WN1	-	-	-	-	-	-	3.38	2.29
LN1	-	-	-	-	-	-	9.2	12.33
WN2	-	-	-	-	-	-	9.9	0.56
LN2	-	-	-	-	-	-	9.4	3.15
WN3	-	-	-	-	-	-	3.38	4.27
LN3	-	-	-	-	-	-	9.2	5.77
WN4	-	-	-	-	-	-	2.04	-
LN4	-	-	-	-	-	-	8.88	-
WN5	-	-	-	-	-	-	0.25	-
LN5	-	-	-	-	-	-	6.04	-
WN6	-	-	-	-	-	-	4.64	-
LN6	-	-	-	-	-	-	4.06	-
Fs	-	-	-	-	-	-	0.32	0.95
Fr	-	-	-	-	-	-	1.62	0.73
YPN	-	-	-	-	-	-	1.18	0.67
YL	-	-	6.25	-	-	-	-	-

**Table 2:** Comparison of simulated results for conventional arrays, arrays in [2], and proposed antenna arrays.

Antenna	Dimension Ground Plane in $\lambda^{2}$	Dimension Patches $\lambda^{2}$	BW	Gain (dB)	Aperture Efficiency	Radiation Efficiency	E-Plane	H-Plane
Preliminary antenna array (2 Elements)	0.47 (1.15λ × 0.41λ)	0.29 (1.03λ × 0.29λ)	$1\%\;(f_{c}\;=$ 5.8 GHz) 5.76–5.83: 70 MHz	7.2	89%	77%	34.3°	88.6°
Proposed Antenna Array (1 Subarray)	0.4 (0.81λ × 0.5λ)	0.26 (0.72λ × 0.37λ)	$5\%\;(f_{c}=$ 5.8 GHz) 5.59–5.91: 320 MHz	7.5	112%	83%	57.6°	86.2°
Proposed Antenna Array *AL (1 Subarray)	0.5 (λ × 0.5λ)	0.36 (0.94λ × 0.39λ)	$12\%\;(f_{c}=\;$5.8 GHz) 5.34–6.07: 730 MHz	8.6	115%	98%	44.9°	86.5°
Conventional Antenna Array (4 Elements)	1.11 (1.14λ × 0.98λ)	0.87 (1.02λ × 0.86λ)	$1\%\;(f_{c}=$ 5.8 GHz) 5.75–5.82: 70 MHz	9.9	70%	80%	35.3°	47.8°
Proposed Antenna Array (2 Subarrays)	0.8 (0.99λ × 0.81λ)	0.61 (0.86λ × 0.72λ)	$5\%\;(f_{c}=$ 5.8 GHz) 5.56–5.9: 340 MHz	9.9	97%	87%	57.5°	51.0°
[2] (Second proposed array) *AL	1.82 (1.52λ × 1.2λ)	1.04 (1.19λ × 0.88λ)	$10\%\;(f_{c}=$ 2.4 GHz) 2.31–2.55: 240 MHz	12.6	84%	96%	-	-
Proposed Antenna Array (4 Subarrays) *AL	1.94 (1.94λ × λ)	1.75 (1.87λ × 0.94λ)	$20\%\;(f_{c}=$ 5.8 GHz) 5.2–6.4: 1.2 GHz	13.9	100%	96%	42.9°	26°
[2] (Third proposed array) *AL	2.88 (1.92λ × 1.50λ)	1.87 (1.6λ × 1.17λ)	$13\%\;(f_{c}=$2.4 GHz) 2.21–2.55: 340 MHz	14.6	78%	96%	-	-
Proposed Antenna Array (6 Subarrays) *AL	2.95 (2.95λ × λ)	2.69 (2.87λ × 0.94λ)	$17\%\;(f_{c}=$ 5.8 GHz) 5.15–6.18: 1.03 GHz	15.5	96%	96%	41.9°	17°

*AL = Air Layer.

## Data Availability

Not applicable.
